# Cross-Modal Effect of Presenting Food Images on Taste Appetite

**DOI:** 10.3390/s20226615

**Published:** 2020-11-19

**Authors:** Keisuke Tomono, Akira Tomono

**Affiliations:** 1 Green Chemistry Centre of Excellence, Department of Chemistry, University of York, York YO10 5DD, UK; keisuke.tomono0829@gmail.com; 2 Department of Information Media Technology, Tokai University, 2-3-23 Takanawa, Minato-ku, Tokyo 108-8619, Japan

**Keywords:** food image, smell, saliva, NIRS, Oxy-Hb, temple, cerebral blood flow, presences

## Abstract

We researched a method to objectively evaluate the presence of food images, for the purpose of applying it to digital signage. In this paper, we defined the presence of food images as a sensation that makes us recognize that food is there, and investigated the relationship between that recognition and the salivary secretion reaction. If saliva secretion can be detected by a non-invasive method, it may be possible to objectively estimate the presence of the viewer from the outside. Two kinds of experiments were conducted. STUDY 1 included presentations of popular cooking images, which portrayed a sense of deliciousness, and evaluated changes in the volume of saliva secretions and cerebral blood flow near the temples. STUDY 2 included comparisons of changes between presenting images only and images with corresponded smells. The images included scenes that introduced foods (i.e., almond pudding cake/bergamot orange) that were relatively simple, so that they did not induce the subjects themselves. As a result, we clarified the cross-modal effects that were closely related to sense of presence and salivation. Moreover, we clarified presentation of images with smells to improve one’s sense of presence, even though the images were relatively simple.

## 1. Introduction

In recent years, researchers have advanced their work on the subject of smell—pertaining to the sense of smell being an element of multimedia, and applying it to the interface of senses (which provides a high sense of presence) [1,2]. Presentations involving the sense of smell are significant, primarily due to its application in various fields, such as movies, games, and digital signage. It is also significant that its application in food advertisements can induce appetite. Smells are known to induce strong effects in humans when it comes to taste and appetite [3]. For example, in everyday life, people often enter restaurants because of the delicious smells. Thus, adding smells to food images displayed on digital signage is expected to have significant effects on advertisements (along with appetite inducement). As described above, food images (with smells) have potential possibilities, but there are also difficulties. These difficulties, for example, may correspond with (i) presenting movies in an environment with a high sense of presence (presence will be discussed further in Section 2.1), which would induce appetite (as if the food was right in front of the viewer); and (ii) evaluating the feelings of the viewer. As for (i), displays that emit smell (closely, from the object displayed on the screen to the viewer) have been proposed [4,5,6]. As for (ii), subjective evaluations using questionnaires have been conventionally used. However, there are difficulties acquiring answers to questionnaires, since it is necessary to ask viewers to focus on the movie during the presentation.

The aim of this research is to consider the possibilities of objective evaluations based on the biological responses regarding the presence of food images. We experience drooling when looking at our favorite foods while we are hungry. This occurs because episodic memories are stimulated due to the sight of food, which can correspond with psychological responses that occur when a person feels motivated to eat [7]. Thus, this research considers observations of the cross-modal phenomenon, where a person experiences taste from sights and smells as inducements. In addition, the research obtained biological responses from participants, related to the emission of saliva when watching movies. One’s sense of presence was subjectively evaluated using a questionnaire and, thus, we focused on certain changes of biological responses related to volume and sensations of saliva [8]. Due to this, we conducted two kinds of experiments, described below.

Section 2 describes the details of the experimental environment, and the measurements of biological responses, which may be useful in understanding saliva secretions and the experimental flow. Section 3 describes the first experiment, called STUDY 1, which presents cooking movies (inducing mouth-watering); the relationship between changes in oxyhemoglobin on the temples and the volume of saliva is also discussed in this section. Section 4 describes the second experiment, called STUDY 2, which tested whether the addition of smells to food images increased the sense of presence. We consider the changes of oxyhemoglobin on the temples and the volume of saliva, and research the sensations involving appetite, saliva secretions, and sense of presence, with questionnaires using food images (which do not induce appetites themselves).

## 2. Experimental Environment and Flow

### 2.1. Factors Involved in Sense of Presence

The sense of presence is defined as the feeling of being there, and it involves two factors: the physical factor, perceived by senses in the real environment; and the sensational factor, generated by memories accumulated through previous experiences [9]. It is necessary to make the physical factor as close to real as possible, in order to increase the sense of presence. By doing so, sensational factors are generated so that a person feels the virtual environment. For this purpose, information regarding sights and smells is presented in the environment where other factors are less likely involved. The evaluation room and experimental equipment are described in detail in the next subsection. Moreover, we had no choice but to ask the subjects themselves about how the internal factors worked. In STUDY 1, the subjects were required to make simple finger movements, according to their appetites, during the experiment. In STUDY 2, a questionnaire was given after the experiment. Details will be described in the experimental methods of each section.

### 2.2. Experimental Room and Method of Stimulating Senses

**Figure 1:**
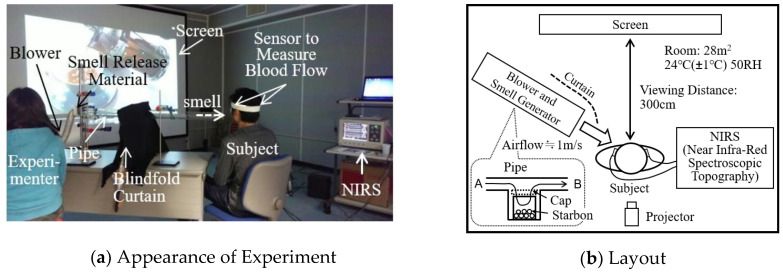
Illustration of the experimental conditions.

The experimental room size was 28 m^2^, and included an air-conditioner, which kept a certain temperature and humidity in the room (and did not allow electromagnetic radiation). In the experiment, we set the room temperature to 24 degrees Celsius and the humidity at 50%, so the subjects could be comfortable. Figure 1 shows the experimental environment. (a) Shows experimental views and (b) shows experimental settings. Images were projected to a large screen (140 inches) using a DLP (Digital Light Processing)^TM^ method projector (NEC Corporation, LT170). The luminosity was 1500 lumen, and the maximum number of pixels (of the display) was XGA (1600 × 1200 px). The range of the image projection was 100 inches, the distance for the sight was 3 m, and the angle for the sight was 37 degrees.

The system of presenting smells, which was used in the experiment for evaluating the images with smells in Section 4, is explained below. Smells were presented to the subjects’ noses, from certain directions that were outside of their range of sight, using an airflow generator (which consisted of a blower and a pipe). The airflow generator was set at the corner of the pipe and the smell-presenting device was set in the middle of the pipe, as described in Figure 1b. As for the smell-emitting materials—Starbons powders (produced at the University of York) were used. The materials have porous structures and are prepared by drying after capturing smells from essential oils [10]; the smells are emitted by directing the airflow towards the materials [11]. As for the tea images, the Starbons (in which essential oils of bergamot oranges were captured) were used. As for the almond pudding cake images, the Starbons (in which essential oils of vanilla were captured) were used. Then, smells were carried by airflow, directed from A to B. The smell levels were adjusted with the amount of Starbons. The airflow speed at the top of the pipe was less than 1 m, and the airflow was directed toward the faces from the side, so the stimulus of airflow was weak.

**Figure 2:**
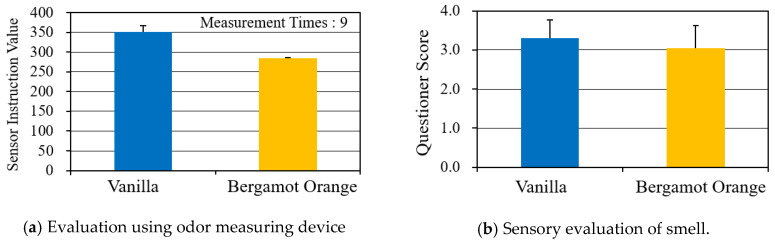
Strength of presented smell.

Figure 2a shows the results of the smell levels, measured by setting a smell sensor positioned 5 cm away from the top of the pipe. The sensor was a new Cosmos Electric Co., Ltd., odor sensor mini, XP-329m [12], which was used to check the relative differences of the smell levels. The averages and standard deviations (SDs) of the data measured nine times for five minutes were calculated. It was confirmed that the smells were released (with stable intensity) when needed due to the small SDs. Figure 2b shows the scent intensity used in STUDY 2—the result of sensory evaluation by 12 subjects. It was adjusted to about level 3, where the kind of smell could easily be perceived in five levels of intensity, from no smells (0) to strong smells (4). Moreover, during the experiment, we made the room dark by turning off the light and used a curtain to keep the light out, in order for the smell-presenting device to be outside of the range of sights.

### 2.3. Device of Measurement for Biological Responses

As for the evaluation of the presence of content, based on the changes of sensational factors generated, along with stimulus provided, the method of comparing the changes (when the actual stimulus was provided) was considered. As for the evaluation of senses, we measured biological responses (while watching content) along with the questionnaires after the experiments. The target of the evaluation was foods images, so we focused on saliva secretion as a biological response [13]. Kamei et al. showed that, by presenting food in front of the subject, subjective appetite and saliva flow increase, while saliva viscosity decreases [14,15]. Furthermore, Sato et al. found that changes of cerebral blood concentration against stimuli of taste, which was measured near the temples, were responsive to the activities of saliva secretion using near infrared spectroscopy (NIRS) [8]. In addition, Matsumoto et al. found that the changes in blood concentration near salivary grands through the stimuli of taste increased due to the presentation of smells [16]. By considering these, if presented food images are perceived with a high sense of presence and mouth-watering, saliva secretion increases, and the change in blood concentration near the temples increases.

As a method of directly measuring the volume of saliva, various methods, such as the spitting method, cotton method, Saxon method, and the gum method are used as well [17]. This experiment aimed to measure volume of saliva while the participants watched movies, so methods of putting materials under the tongue, and chewing, were not suitable. In order to reduce uncomfortable feelings in the participants’ mouths (as much as possible), we adopted a modified Watts method [18]. Concretely, we used a saliva absorption sheet, with a rough size of 2 mm thick and 4-cm^2^ large (Salivatol by OJI KINOCLOTH Corporation). This sheet was made of cellulose, mixed with polyester polyethylene composite fiber. It is excellent at saliva absorption and water retention capacity, as well as in stability in the oral cavity. It has little stickiness, which makes us feel uncomfortable. The volume of saliva absorbed by the sheet was accurately measured by a high-precision weight scale (a sonic type analytical scale by Shinko Denshi Corporation: HTR-220), with the minimum weighing capacity of 0.1 mg.

A near infrared oxygenation monitor (NIRO-200) was used to measure cerebral blood flow [19]. The principle of the measurement was based on the near infrared spectroscopy (NIRS) [20]. Near infrared light (wavelength 700 to 900 nm) was irradiated into the skull, and attenuated light, after moving the brain at a predetermined distance, was captured by the light receiving sensor. The near infrared LED and the light receiving sensor were configured together as a NIRS sensor device; it was mounted and used in a place to examine the brain activity of the head. As the living body is a strong scattering medium, a presence of a light absorbing object, such as hemoglobin on the travel route of light, weakens the light, due to the Beer–Lambert Law. Therefore, by analyzing the light sensor output, it was possible to convert it into the blood flow amount. The distance of sensors in the device was 4 cm, and changes of cerebral blood flow about 1 cm away from the surface of the brain (about 3 cm away from the skin surface) were measured. As for the method of measuring the changes of cerebral blood flow, the modified Beer–Lambert method [21] and spatially resolved spectroscopy (SRS) method [22] were used. The changes of oxyhemoglobin (Oxy-Hb), deoxyhemoglobin (Deoxy-Hb) concentrations, and Tissue Oxygen Index (TOI) were measured. Since the distance of light from irradiation to detection cannot be measured, the obtained results are the changes of relative values of Hb concentration, not absolute values.

### 2.4. Experimental Preparation and Flow

**Figure 3:**
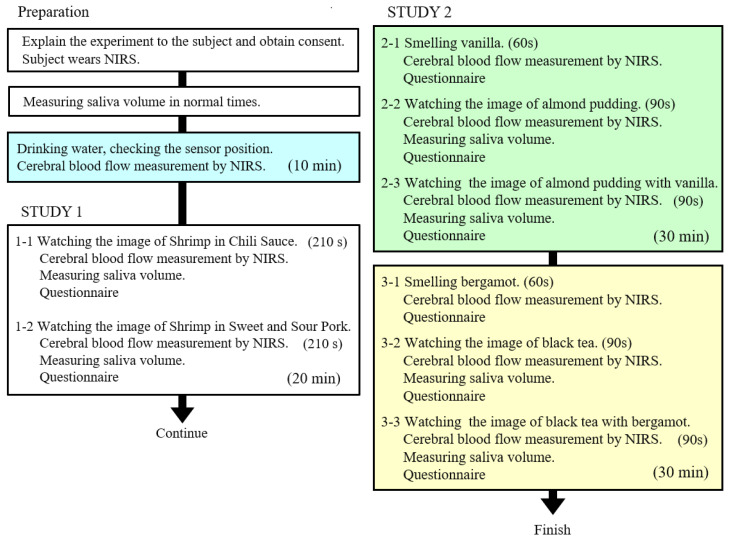
Experimental procedure for 12 subjects whose saliva volume and cerebral blood flow changes were measured simultaneously. (Apart from this experiment, an experiment was conducted on 10 subjects to measure only cerebral blood flow without simultaneously measuring saliva).

Figure 3 shows the whole experimental flow. The experimental concept was explained to the subjects, and subjects were asked to fill out the approval forms. Saliva volumes were measured in their normal conditions (as a preliminary experiment), and subjects were asked to attach the NIRS sensors. Herein, the methods of measuring saliva and attaching NIRS sensors are described.

**Figure 4:**
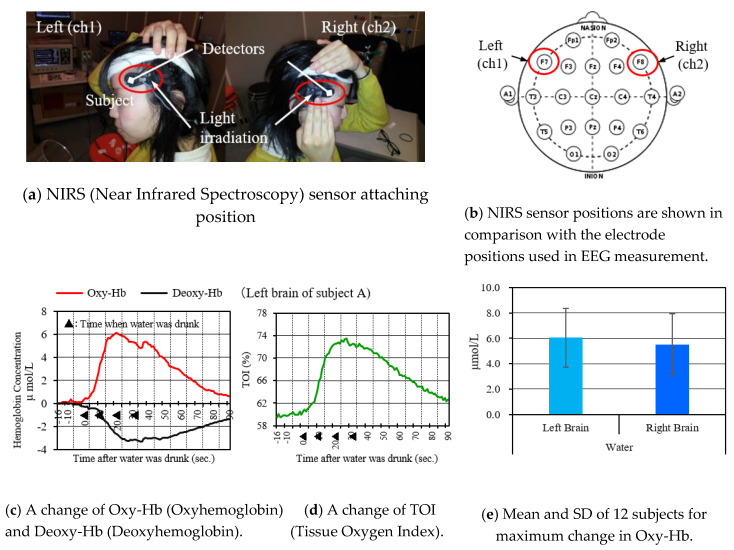
NIRS sensor attaching position and reaction when water was drunk.

(1) Measuring saliva: the saliva volumes, in normal condition, differ by person. It has to be checked in order to understand the changes in saliva when watching images. Therefore, a sheet was placed in a mouth for about five minutes and then taken from the mouth. The saliva volumes were calculated as the volume, for five minutes in the normal condition, by subtracting the weight of the sheet itself from the sheet including saliva. The images used in STUDY 1 and 2 differed in length, and the saliva volumes were modified to fit the length of the images, and calculated as the volume in normal condition at that time. After that, the saliva volumes were measured by using the same method when watching images; the difference with the volume in normal condition was calculated as a change in saliva when watching images.
(2) Method of attaching NIRS sensors: as Figure 4a shows, the NIRS sensors were attached in temples. Sato et al. used multiple NIRS sensors to clarify the sensor position, which effectively measured the responses by saliva during eating [8]. We referenced their findings for our study. By referencing the ten twenty electrode system of the electroencephalograph attaching positions, these sensor positions corresponded to the positions indicated by the red circle in Figure 4b. However, there were differences, by person; the sensor positions were modified by conducting a preliminary experiment in each person. When a person eats, saliva is secreted, so we asked the subjects to drink 20 cc of water in a cup to measure the change of outputs. Figure 4c shows the changes in Oxy-Hb and Deoxy-Hb attached to the targeted position. Figure 4d shows Tissue Oxygen Index (TOI). The ▲ symbol in the figure indicates putting water in a mouth, and generating secretion of saliva. It is understood that these sensor values were significantly changed. From the response of the NIRS, the following can be considered: when the brain is activated, oxygen is consumed, so, along with consumption, new blood (which contains oxygen) is compensated into the parts. Therefore, Oxy-Hb (of the position) increased, while Deoxy-Hb (of the position) decreased. Oxy-Hb and Deoxy-Hb have corresponding changes; Oxy-Hb was to consider the state of brain activation.

Figure 4e shows the maximum changes of Oxy-Hb in each subject and the results, which calculate the averages and standard deviations. We checked if the responses were obtained from all of the subjects (in the conditions of secreting saliva by eating).

Next, two kinds of experiments were conducted. STUDY 1 and STUDY 2 focused on the increase of saliva along with the presence and responses of NIRS sensors corresponding to the responses. Both studies have common experimental purposes that research cross-modal effects. However, as for the research concerning presence (by presentation of smell), the experimental purposes differ. Moreover, the presentation images differed. The details of the experimental method and results are explained in Section 3 and Section 4.

By conducting experiments in the flow stated in Figure 3, the time for each subject took about one and half hours. Rest was set between the experiments in order for participants to have better concentrations on the experiments.

### 2.5. Participants

The research evaluates the presence with the measurement of physiological response using human subjects. We obtained approval from the ethics committee regarding the use of a human as a subject in Tokai University, and conducted research on the subjects after obtaining informed consents.

The subjects were 22 Japanese university and graduate students (12 males/10 females), healthy, between the ages of 21 and 24 years old, and whose eating habits and culture were generally the same. We asked them to make sure that their conditions were fine, and that they did not take medication, because it may have caused them to become sleepy during the experiments. Furthermore, we asked them not to eat anything at least two hours before the experiments.

Herein, the relationship between the number of subjects and the experiments is described. Among the flows in Figure 3, the experiment, which measures the saliva volumes and NIRS at the same time, caused some difficulties on the subjects, and were not easy. The number of subjects who participated in this experiment in this flow was 12 (6 males/6 females). In addition, there were 10 subjects who participated in this experiment without measuring saliva volumes. In this paper, most of the results dealt with 12 subjects, but the NIRS responses in STUDY 2 dealt with a higher number of data in order to increase the reliability of statistics.

## 3. Psychological Effects and Cooking Images (STUDY 1)

### 3.1. Purpose

If we watch a cooking image and feel that there is food there, that is, if the presence is high, saliva production will increase. If this cross-modal phenomenon is associated with brain activity measurement, it can be used for objective evaluation of presence. Therefore, we conducted a psychological evaluation using a popular cooking image that induced hunger by presenting delicious-looking food, and focused on which scene in this image enhanced the sense of presence, and caused the NIRS reaction related to the saliva secretion reaction.

### 3.2. Contents of Images That Induced Hunger by Presenting Delicious-Looking Food

**Figure 5:**
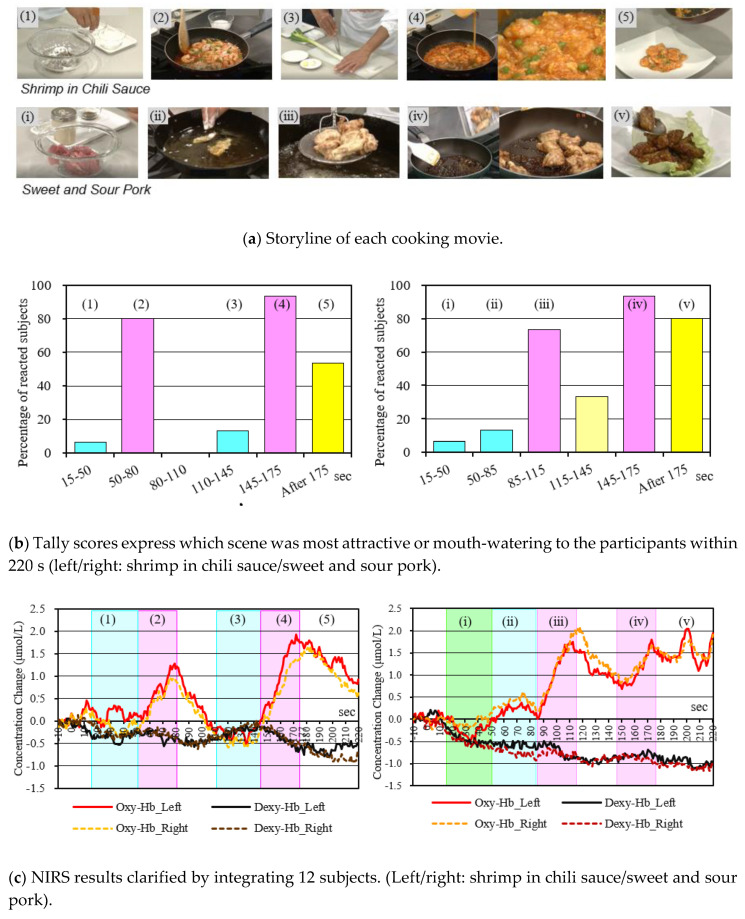
NIRS results when the subjects were conscious of wanting to eat in each cooking movie of STUDY 1; (**1**) and (**3**)/(**i**) and (**ⅱ**) cycles show preparatory scenes, such as seasoning and shaping ingredients, and (**2**) and (**4**)/(**ⅲ**) and (**ⅳ**) show sizzling-simmering scenes.

As shown in Figure 5a, two movie clips involving cooking were designed by editing official images from Japanese TV cooking programs [23]. The movies depicted cooking of Szechuan-styled cuisine, shrimp in chili sauce, and sweet and sour pork, and the process was based on sizzling-simmering, with the smell seeping into the air above. The visuals were designed to show two contrasting cooking scenes: “braising over sizzling fire” and “preparing condiments”,—the former giving stronger brain stimulation.

#### 3.2.1. Shrimp in Chili Sauce

The following are the sequential (time-wise) steps involved in preparing this dish: 15 to 45 s for seasoning the shrimp (squishing with cooking potato starch), 50 to 80 s for sizzling the shrimp, 110 to 140 s for shaping green onions (chopping), and 145 to 175 s for simmering the shrimp with eggs and onions.

#### 3.2.2. Sweet and Sour Pork

The following are the sequential (time-wise) steps involved in preparing this dish: 15 to 45 s for seasoning the pork (coating with potato starch), 50 to 80 s for simmering in oils, 85 to 115 s for preparing the flavoring sauce (mixing), and 145 to 175 s for sizzling the pork in the sauce.

Since the subjects were in the same food culture area, these cooking contents were fully understandable. In addition, it was confirmed before the experiment that participants had experiences of eating these types of dishes and the dishes were their favorite foods.

### 3.3. Experimental Method

As shown in Figure 3, the subjects were asked to watch the above two types of images while having the saliva absorption sheet (described in Section 2.4) in their mouths. While watching the images, the subject’s cerebral blood flow data were measured in real time. They were also instructed to move their finger slightly to inform the experimenter when they had a strong saliva sensation. The experimenter behind the subject confirmed this sign and recorded it, along with the cerebral blood flow data. After viewing, saliva volume was measured by the method described in Section 2.4. We also conducted a questionnaire about the impression of the image. The two types of images were presented in random order for each subject.

### 3.4. Experimental Results

**Figure 6:**
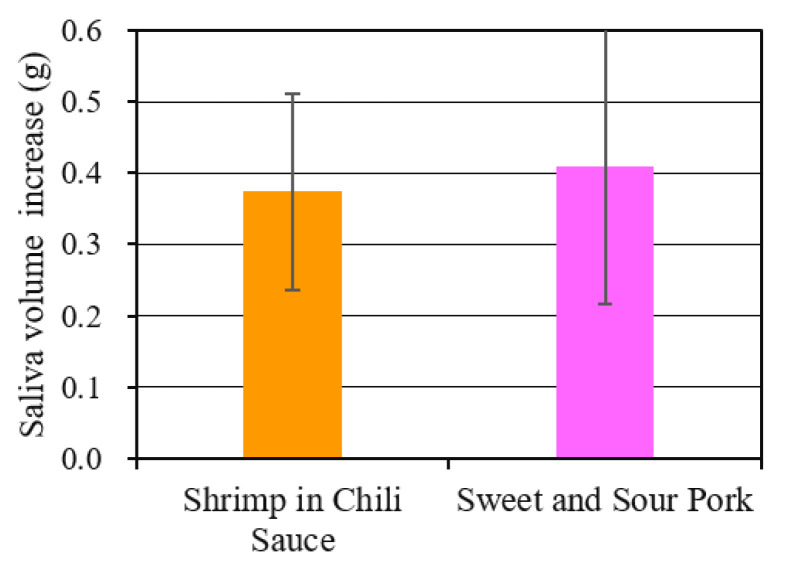
Changes in saliva volume after viewing contents in STUDY 1.

Figure 5b shows the results of arranging the ratio of the subjects who signed with their finger for each scene. It can be seen that many subjects experienced a feeling of saliva in a scene where meat was roasted or was likely to have a strong scent. Figure 5c shows the change over time of the Oxy-Hb concentration and the Deoxy-Hb concentration obtained from the left and right sensors attached to the subject. This is the result of averaging the data for 12 subjects at each time. It is possible to know, in which scene, the subjects’ brains were activated, on average. In Figure 5b, an increase was observed in the scene where a strong saliva sensation was generated. Many subjects felt that the presence of the image was generally high. Figure 6 shows the mean values and standard deviations as a result of examining the change in saliva volume due to image viewing. The mean values increased by about 0.4 g in each movie. In each set, the null hypothesis was that there was no increase in saliva volume, and when (one-sample) *t*-tests were performed, the hypothesis was rejected, and an increase in saliva volume was confirmed at a significance level of 5%.

### 3.5. Discussion

The NIRS sensor detected a brain activation reaction in which the Oxy-Hb concentration increased when the subject saw the immersive scene (in which he felt saliva secretion). Therefore, the NIRS sensors attached to the temples captured the cross-modal phenomenon (that visual stimuli stimulated the sense of taste); it is believed to be useful for examining the presence of food images. Considering this, together with the results of the questionnaires, in a series of scenes, there was a tendency to react strongly in the scene that imagined eating, such as grilling meat and adjusting the taste. It is imagined that the brain was strongly influenced by the content and felt like being in the kitchen.

As reported by Kamei et al. [14], saliva has a different viscosity depending on the psychological state, and is high in viscosity when it is tense, and low in viscosity when it is relaxed, such as during a meal. In this experiment, only saliva volume was measured, not viscosity. In the future, if the autonomic nervous system is measured at the same time, it may become clearer what the saliva secretion means.

## 4. Psychological Effects and Cooking Images (STUDY 2)

### 4.1. Purpose

We perceive food scents through our five senses. Therefore, although appetite is not simulated solely by photos and images, when there is a scent, the food suddenly seems realistic and the appetite improves. In reference to these experiences and the results of Section 3, we investigated how the addition of smells to images affected the appetite and sense of presence. Smell is a medium that recalls episode memories. In order to emphasize the effect of the smell more, we used fairly simple images. That is, in the content production, when the image and the smell were presented in an integrated manner, it was expected that the previous eating experience would be recalled and the sense of presence would be enhanced. We measured changes in saliva volume and oxygenated hemoglobin concentration near the temples, along with the Kansei evaluation questionnaire regarding the sense of presence.

### 4.2. Content for Investigating Smell Effects

**Figure 7:**
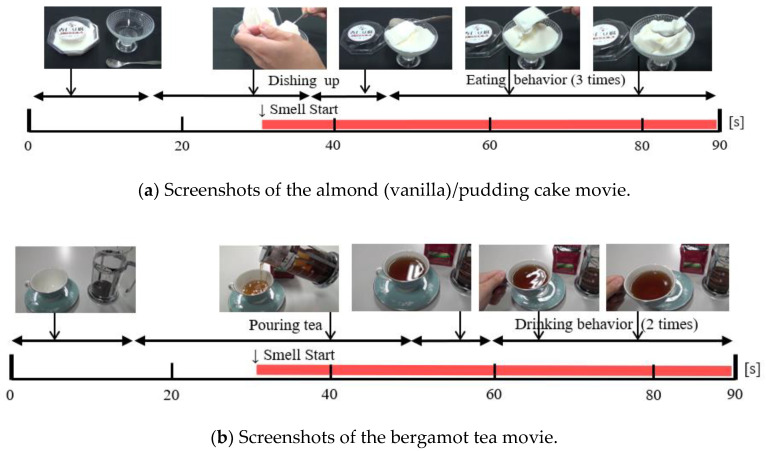
Storylines of food movies for STUDY 2.

For the experiment, the images of almond pudding cake and bergamot tea (Darjeeling tea) shown in Figure 7 (and produced by us) were used. Both are popular foods with characteristic smells, and the smell was believed to affect a participant’s motivation to eat. The former was selected as an example of sweets and the latter as an example of drinks. The story of the former consisted of scenes (of about 90 s) of taking almond pudding cake out of a plastic container, placing it on glass tableware, and trying to eat it. The latter consisted of scenes of about 90 s: placing leaves in a roasting container, pouring hot water, pouring bergamot tea into a tea cup, and trying to drink it. In both cases, the first 30 s did not include scenes that made the viewers strongly aware of the smell. The middle 30–60 s included scenes where the smells would have been recognized if it were in the real world. The latter 60–90 s included scenes where the food was about to be eaten, so, if episodic memory worked, it was easy to recall the smell. The images as a whole had a light and slightly subdued impression, and did not actively induce food intake.

### 4.3. Experimental Method

In the experiment, as shown in Figure 3, the contents related to almond pudding cake and bergamot tea were presented under the three conditions of “Smell” for the smell only, “Image” for the image only, and “Image + Smell” for the image with smell, respectively. NIRS data of subjects watching the content was acquired in real time.

The main purpose of this experiment was to compare “Image” and “Image + Smell”. “Smell” was added to consider the effect of smell on “Image + Smell”. The time for the smell to be presented in “Image + Smell” was 60 s. Therefore, the presentation time of “Smell” had been adjusted accordingly. In comparing “Image” and “Image + Smell”, it was necessary to consider the possibility that the previous experiment would bias the later experiment. To solve this problem, a method of canceling the bias by repeatedly presenting these stimuli to the same subject in a random order was considered, but in that case, there was concern that new problems would arise, such as an increase in the burden on the subjects and habituation due to the learning effect. Therefore, the 12 subjects were randomly divided into two groups of six, and experiments were conducted in which the presentation order was different. The contents were presented to the first group member in the order of “Image” to “Image + Smell”, and the second group member in the order of “Image + Smell” to “Image”. Each subject viewed the two types of content once. The data obtained were processed together.

For “Image” and “Image + Smell”, saliva volume was measured using the method described in Section 2.4.

The questionnaire after the experiment was conducted, as follows. In this experiment, the presence of food images was defined as “feeling as if there was food in front of us”, and images related to the foods that the subject liked were used. Therefore, a Kansei evaluation questionnaire consisting of four items, asking about saliva sensation (feeling of saliva coming out), appetite (motivation to eat), presence (feeling of food being present there), and episode (feeling of recollecting past eating experience), was conducted. Each item was scored on a 5-point scale from not felt (0 points), moderate (2 points) to strongly felt (4 points).

### 4.4. Experimental Results

#### 4.4.1. Questionnaire Results

**Figure 8:**
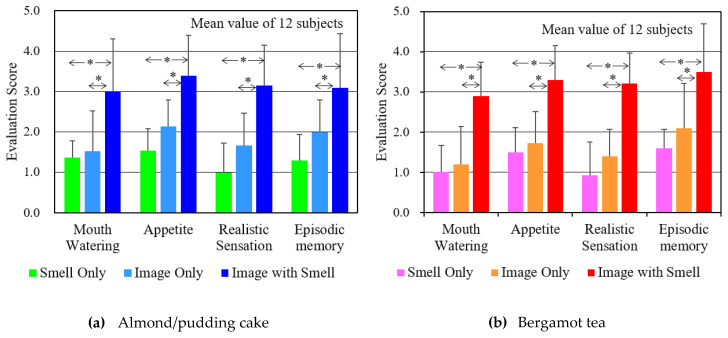
Subjective evaluation score upon feeling “mouth-watering”, “Appetite”, “Realistic Sensation”, and “Episodic Memory” for estimating the sense of presence when watching food images.

Figure 8a,b show the mean value and standard deviation of the scores of the subjects for the almond pudding cake content and the bergamot tea content, respectively. For each evaluation item, “Image + Smell” was obtained as a result of feeling “quite think so” (around 3 points). In order to compare the results of “Image + Smell” with “Smell” or “Image”, differences were tested for mean scores. Since the standard deviation was different, when using the t-test of Welch, the null hypothesis (that there was no difference) was rejected at a significance level of 5%, the difference between the two became clear, as shown by (*) in the figure. Furthermore, when the impression of the experiment was investigated by free answers, many subjects said, “When I felt appetite and saliva, I remembered the taste of the food I had eaten before”, and “In the image with smell, the smell perception was a clue to remembering episodic memory”.

#### 4.4.2. Saliva Volume

**Figure 9:**
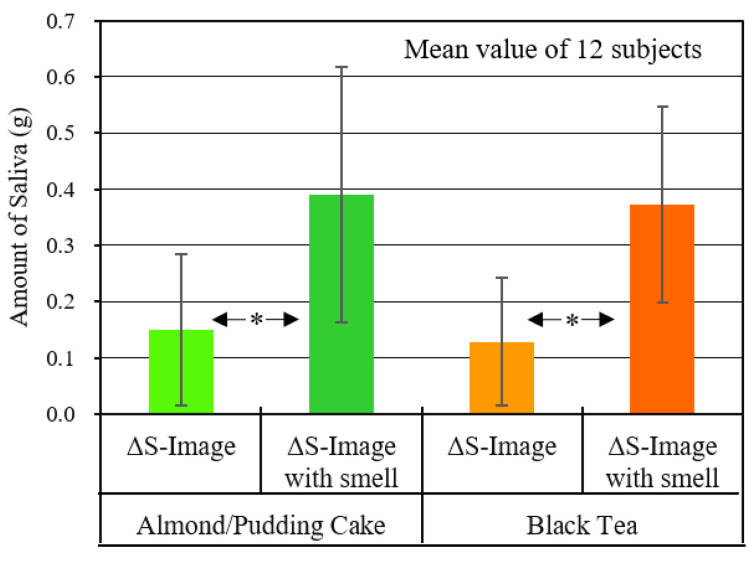
Changes in saliva volume after viewing content (ΔS) in STUDY 2.

Figure 9 shows the difference between the volume of saliva secreted during the viewing time for “Image” and “Image + Smell” and the volume of saliva secreted at the same long time of 90 s in normal time, described in Section 2.4. This is the result of calculating the mean value and standard deviation of the subjects.

As for the results of almond pudding cake and bergamot tea, it was found that the mean values were positive under all conditions, and the volume of saliva increased compared to normal times. Moreover, as a result of testing the difference between the mean values of “Image + Smell” and “Image” using the t-test of Welch, it was found that “Image + Smell” was higher at the significance level of 5% for both almond pudding cake and bergamot tea.

#### 4.4.3. Change in Oxygenated Hemoglobin Concentration

**Figure 10:**
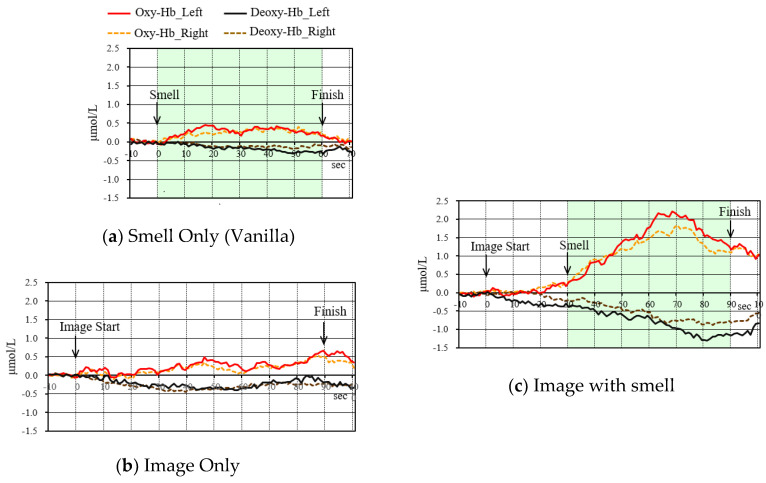
NIRS results of the almond/pudding cake study, clarified by integrating 12 subjects. (Solid lines/dotted lines: left/right temple regions). (Red/black: Oxy-Hb/Deoxy-Hb).

**Figure 11:**
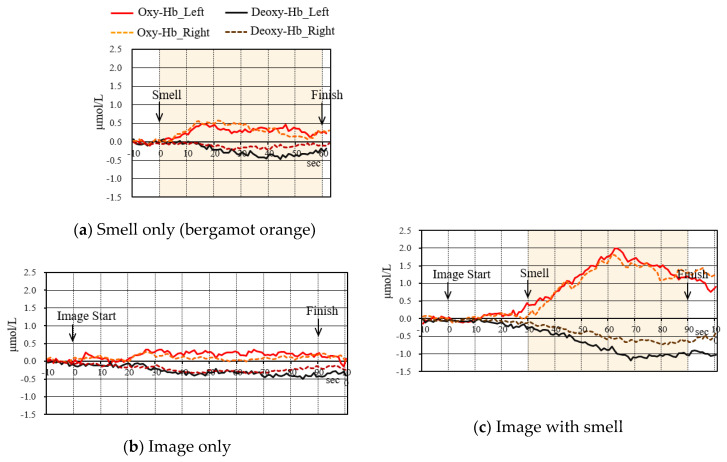
NIRS results of the bergamot tea study, clarified by integrating 12 subjects. (Solid Lines/Dotted Lines: Left/Right temple regions). (Red/Black: Oxy-Hb/Deoxy-Hb).

Figure 10 and Figure 11 are the results of oxygenated hemoglobin concentration when the almond pudding cake content and bergamot tea content were presented under the conditions of “Smell”, “Image”, and “Image + Smell”, respectively. Mean changes in 12 subjects are shown, as a result of performing the same processing, as described in Figure 5c. In “Smell” and “Image”, the response of the subject was small as a whole, and the response scene differed, depending on the subject, so that the value was smoothed by the averaging process, and became a small value. On the other hand, in “Image + Smell”, the value was similar to the value of “Image” before adding the smell, but it became a large value because many subjects responded similarly to the start of the smell. In particular, it became the largest in the scene to eat.

**Figure 12:**
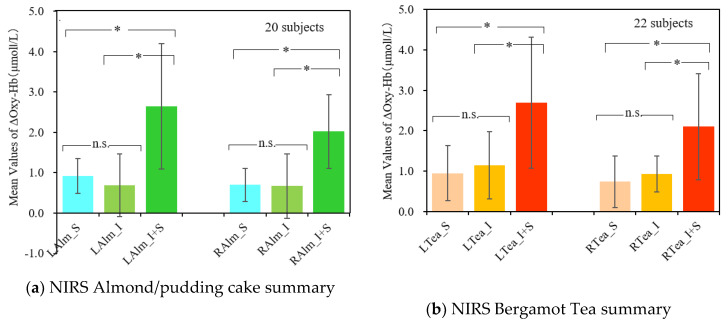
Summary of ΔOxy-Hb response. LAlm_S illustrates the study of almond pudding cake with smell presentation on the left side, and RTea_I + S the study of bergamot tea with Image + Smell presentation on the right. In order to improve the accuracy of the statistics, we also used data from a group of subjects who measured only cerebral blood flow, without simultaneously measuring saliva. (Asterisk * denotes significant/n.s. denotes non-significant).

Figure 12 summarizes the change value of the oxygenized hemoglobin concentration (ΔOxy-Hb reaction) compared to the contents using the data set of these figures. (a) Was an image related to an almond pudding cake, and (b) was a bergamot tea related image. ΔOxy-Hb reaction was calculated as follows. For each subject, the average value of the oxygenation hemoglobin concentration, 30 s before the content presentation as a baseline, the change value of the section corresponding to the scent presentation was obtained in each measurement cycle. Following this, the average of the entire subject was calculated. That is, the ranges of calculating averages were 0 to 60 s in smell, 30 to 90 s in image, and 30 to 90 s in image and smell. All of the ranges were for 60 s. As described in Section 2.5, 22 subjects participated in this experiment. It can be considered that, the more dataset there is, the more reliability. Thus, for almond pudding cake, the data of 20 subjects (excluding the data of two subjects whose data were failed to obtain) were used for the analysis, while for bergamot tea, the data of 22 subjects were used for the analysis.

The validation of data was confirmed by the Shapiro–Wilk analysis, except for LAlm_I + S and RTea_I + S. Additionally, the data that failed the validation of the analysis were confirmed by the interpretation of visuals of qq plots and histogram. Thus, there was no significant reason to deny the validation, and the use of the parametric model of statistics was not considered to have a significant risk. Thus, applying repeated measurement analysis of variance (ANOVA) indicated that both almond pudding cake and bergamot resulted in the significance level of below 1% among (S), (I), and (I + S) for each of left and right region. Pairwise Comparative analysis upheld the significances on (LAlm_I + S) and (RAlm_I + S), as below 1% for all the comparisons. Therefore, the images with smells had higher averages of Oxy-Hb than images or smells only, which lead to the activation of brain and increase of the presence.

### 4.5. Discussion

In the experiments using two different types of content, almond pudding cake and bergamot tea, the following common characteristics were found. Since these images were merely to introduce how to eat the foods, and were different from the images that induced hunger by presenting delicious-looking food described in Section 3, the subjects did not give a high score to saliva feeling, feelings to eat, presence, or episodic memory in the questionnaire. Saliva volume did not increase much either. Moreover, the increase in Oxy-Hb concentration was slight. On the other hand, when the smell was presented, the numerical values of all these items improved.

As shown in Figure 4a, the location where the NIRS sensors were attached were slightly above the temple and close to the dorsolateral prefrontal cortex. This area is said to be related to memory, cognition, self-control, motivation, and judgment [24,25,26,27]. With reference to these, it can be considered that the change in these measured values was caused by the increased motivation to eat because the episodic memory (from when one ate in the past) was recalled by the image with smell, and the food image was perceived with a higher sense of presence [28].

## 5. Conclusions

Focusing on saliva secretion and the presence of food images, we measured saliva volume and oxygenated hemoglobin concentration near the temples, and clarified the following.

(1) When participants watched popular cooking images, saliva volume tended to increase during content where participants had motivation to eat or a sense of presence. At this time, a phenomenon was observed in which the oxygenated hemoglobin concentration, which indicates the brain activity level near the temples, increased.
(2) Even if the content did not motivate participants to eat with images alone, adding an appropriate smell increased the motivation to eat and presence. At this time, a phenomenon was observed in which the volume of saliva increased and the oxygenated hemoglobin concentration near the temples increased.

As described above, for food images, there is the possibility that biological response measurement can be used for objective evaluation of presence. However, in this experiment, the reverse verification that saliva is always produced when the oxygenated hemoglobin concentration near the temple increases, was not performed, so further research is required. For example, estimation accuracy is expected to improve when combining it with a user’s target gaze information obtained from an eye camera [29]. In the future, if real-time evaluation of feelings becomes possible using biological response measurement, various applications, such as interactive content development, are expected.
